# Supplementary material for: Hepatitis B immunity and vaccine completion among adults at increased risk for hepatitis B infection in Zambia
Source: PLoS One. 2026 Feb 26;21(2):e0339690. doi: 10.1371/journal.pone.0339690 (PMC12944761; doi:10.1371/journal.pone.0339690)
Supplement: S1 Table — (DOCX) [file pone.0339690.s001.docx]

| **Supplementary Table 1. Correlates of isolated hepatitis B core antibodies among at-risk and surface antigen-negative adult populations in Zambia** | | | | | |
| --- | --- | --- | --- | --- | --- |
|  | # (%) with isolated core | Bivariable | | Multivariable* | |
|  |  | Odds ratio | P value | Odds ratio | P value |
| Age, in years  18-39  40+ | 23 (6.3)  36 (18.9) | Reference  3.45 (1.98-6.03) | <0.001 | Reference  2.14 (1.20-3.83) | 0.01 |
| Sex  Women  Men | 32 (9.4)  27 (12.6) | Reference  1.40 (0.81-2.41) | 0.23 |  |  |
| Population  HCWs  Contacts  PLWH | 2 (1.1)  44 (14.3)  13 (20.3) | Reference  15.31 (3.66-63.94)  23.32 (5.10-106.72) | <0.001  <0.001 | Reference  11.95 (2.82-50.68)  15.37 (3.24-72.85) | 0.001  0.001 |
| *Factors associated with the outcome at P<0.2 in bivariable analysis were included in the multivariable model. Abbreviations: HBV, hepatitis B virus; HCW, healthcare worker; PLWH, person living with HIV infection. | | | | | |
