# Supplementary material for: Hepatitis B immunity and vaccine completion among adults at increased risk for hepatitis B infection in Zambia
Source: PLoS One. 2026 Feb 26;21(2):e0339690. doi: 10.1371/journal.pone.0339690 (PMC12944761; doi:10.1371/journal.pone.0339690)
Supplement: Attachment — Submitted filename: Response to reviewers.docx [file pone.0339690.s003.docx]

Kindly find responses to reviewers.

1. Please ensure that your manuscript meets PLOS ONE's style requirements, including those for file naming. The PLOS ONE style templates can be found at

<https://journals.plos.org/plosone/s/file?id=wjVg/PLOSOne_formatting_sample_main_body.pdf> and

[https://journals.plos.org/plosone/s/file?id=ba62/PLOSOne_formatting_sample_title_authors_affiliations.pdf](https://track.editorialmanager.com/CL0/https:%2F%2Fjournals.plos.org%2Fplosone%2Fs%2Ffile%3Fid=ba62%2FPLOSOne_formatting_sample_title_authors_affiliations.pdf/1/010f01980efb09c3-8cbe96a8-6cc4-4011-98ab-3ed9e1e6eacb-000000/K5EljNqLaIeq64eqYnfuKsoE04mLgATiMOEAFN6ggcE=220).

Corrections to better align with PLOS ONE style template have been made.

2.  We note that the grant information you provided in the ‘Funding Information’ and ‘Financial Disclosure’ sections do not match.

When you resubmit, please ensure that you provide the correct grant numbers for the awards you received for your study in the ‘Funding Information’ section.

We now included the following: “The data collected in this article were generated through two grants from the U.S. National Institutes of Health (R01AI148049 and R37AI179640).”

3.  Thank you for stating the following financial disclosure:

 [This study was supported by grants from the U.S. NIH, grant number

 R01AI148049 received by DLT and grant number R37AI179640 received by MJV.].

Please state what role the funders took in the study.  If the funders had no role, please state: ""The funders had no role in study design, data collection and analysis, decision to publish, or preparation of the manuscript.""

If this statement is not correct you must amend it as needed.

Please include this amended Role of Funder statement in your cover letter; we will change the online submission form on your behalf.

The statement "The funders had no role in study design, data collection and analysis, decision to publish, or preparation of the manuscript." Shall be added to the financial disclosure section. The grant number for MJV was also corrected R37AI179640.

4. In the online submission form, you indicated that [Data is available on request due to ethical restrictions.].

All PLOS journals now require all data underlying the findings described in their manuscript to be freely available to other researchers, either 1. In a public repository, 2. Within the manuscript itself, or 3. Uploaded as supplementary information.

This policy applies to all data except where public deposition would breach compliance with the protocol approved by your research ethics board. If your data cannot be made publicly available for ethical or legal reasons (e.g., public availability would compromise patient privacy), please explain your reasons on resubmission and your exemption request will be escalated for approval.

This data has been made available on figshare.

5. We note that you have included the phrase “data not shown” in your manuscript. Unfortunately, this does not meet our data sharing requirements. PLOS does not permit references to inaccessible data. We require that authors provide all relevant data within the paper, Supporting Information files, or in an acceptable, public repository. Please add a citation to support this phrase or upload the data that corresponds with these findings to a stable repository (such as Figshare or Dryad) and provide and URLs, DOIs, or accession numbers that may be used to access these data. Or, if the data are not a core part of the research being presented in your study, we ask that you remove the phrase that refers to these data.

The data will be added as a table in the supplementary section.

6.  Please include your full ethics statement in the ‘Methods’ section of your manuscript file. In your statement, please include the full name of the IRB or ethics committee who approved or waived your study, as well as whether or not you obtained informed written or verbal consent. If consent was waived for your study, please include this information in your statement as well.

A full ethics statement has been made in the methods statement.

7. If the reviewer comments include a recommendation to cite specific previously published works, please review and evaluate these publications to determine whether they are relevant and should be cited. There is no requirement to cite these works unless the editor has indicated otherwise. 

5. Review Comments to the Author

Reviewer #1: In this manuscript, the authors describe Hepatitis B seroprevalence in adults living in Zambia and tested longitudinally the anti-HBs antibody titers detected at least 1 month after a single vaccination dose.

The study extends our knowledge of HBV incidence in African population and shows that the presence of anti-HBc antibody is associated with a rapid increased of anti-HBs titer after a single vaccination, likely caused by the presence of helper T cells specific for HBs in this population.

I have only a minor request related to the data and some general considerations about the background information and the implications of some results.


Results: Table 3 Anamnestic response

The data presented are of interest but the results lack a control since it will be important to show that individual without anti-HBc have no anamnestic response.

This review suspects that in hyperendemic area many individuals might actually have anamnestic response even in the absence of anti-Hbc antibodies, mainly due to the fact that the the accelerated level of ani-HBs production after vaccination is likely due to the existence of CD4 T cells ( specific for core or envelope) that might be present even in the absence of antibodies. In any case, the authors should show the incidence of rapid anamnestic response in individuals who are not anti-HBc+.

**Our response**: Thank you for the comment. Unfortunately, we did not assess the development of antibodies in people who were anti-HBc-negative. And we only assessed the development of antibodies following a single vaccine dose. However, in another study of health workers in Zambia who received HBV vaccine in the absence of core antibodies, ~20% developed anti-HBs >10 mIU/ml after the first dose of vaccination this increased to 100% after the third dose. This statement has been added to the discussion.

Having said this, the possibility that antibodies anti-HBc might not be able to define all the individuals who encounter HBV in their life time warrants discussion One of the main concern is that this manuscript focuses solely on antiviral immunity in humans as being primarily based on antibody response. However, it is evident that memory antiviral-specific immunity includes both humoral (antibody-mediated) and cellular (T cell-mediated) components. The recent COVID-19 pandemic has clearly shown that healthy individuals can contract SARS-CoV-2 without developing a robust antibody response. Nevertheless, these individuals often exhibit an anamnestic T cell response specific to SARS-CoV-2 (see for example Samandari, T. et al. Prevalence and functional profile of SARS-CoV-2 T cells in asymptomatic Kenyan adults. J. Clin. Investig. 133, (2023).). This possibility was also showed for other viral infections ( see 1.Mok, C. K. P. et al. T-cell responses to MERS coronavirus infection in people with occupational exposure to dromedary camels in Nigeria: an observational cohort study. Lancet Infect Dis 21, 385–395 (2021). 1.Zhao, J. et al. Recovery from the Middle East respiratory syndrome is associated with antibody and T cell responses. Sci Immunol 2, eaan5393 (2017).).

This reviewer is aware that the possibility that people would only mount HBV-specific T cells in the absence of anti-HBV antibodies has not been studied yet in HBV infection, and I am also not certainly asking to analyse the HBV-specific T cell response. However, the possibility that serology might not be sufficient to reveal all the past history of exposure to HBV should at least be mentioned and discussed.

**Our response:** We agree with the reviewer that the development of anti-HBs does not provide comprehensive assessment of immunity to HBV and the anamnestic response. In the revised manuscript discussion section, we added as a limitation that serology does not fully characterize the situation.

There are other points that need to be properly discussed: HBV, like any other DNA virus, is never completely eliminated and as such, the lack of detection of HBV-DNA in the serum is certainly not a demonstration that HBV is not present in these individuals. We know that patients who experienced acute HBV infection still harbour HBV-DNA in their liver that is detected only by analyzing liver biopsies ( see Michalak, T. I., Pasquinelli, C., Guilhot, S. & Chisari, F. V. Hepatitis B virus persistence after recovery from acute viral hepatitis. Journal of Clinical Investigation 94, 907 (1994). Rehermann, B., Ferrari, C., Pasquinelli, C. & Chisari, F. V. The hepatitis B virus persists for decades after patients’ recovery from acute viral hepatitis despite active maintenance of a cytotoxic T-lymphocyte response. Nature Medicine 2, 1104–1108 (1996).)

This fact should be clearly discussed in the introduction. A further consequence of HBV persistence is the real benefit of vaccination in individuals who are already anti-HBc+ ( and as such likely infected by HBV and harbouring HBV-DNA). A recall vaccination might not be important in these individuals to protect them from infection since they might already carrier the virus. Vaccination in these indivduals can indeed boost anti-HBs antibody production and this might be important to help individuals better control the infection or avoid possible re-infection from different HBV strains (??) . We know that we don’t have the answer to these questions, but such a possibility should be discussed. HBV pathogenesis is complex and need to be properly represented.

**Our response:** These are important points. In the revised introduction, we added additional information in the discussion including the point that core antibodies suggest that some hepatocytes may contain HBV nucleic acids. In the revised discussion we point out that vaccination of people without anti-HBs can help avoid superinfection.

Reviewer #2: This is a relevant study addressing an important public health gap in Zambia. The study provides valuable real-world data on HBV seroprevalence, isolated anti-HBc profiles, and vaccine uptake in a sub-Saharan African setting where adult HBV immunization has historically received limited attention.
To strengthen the manuscript, I recommend the following specific revisions:

Introduction:

1 - I recommend that the authors include detailed background information on Zambia’s national HBV vaccination schedule. Please specify the target age groups, whether adult vaccination is currently available and free of charge, the estimated national coverage rates (both for children and adults, if available), and the types of HBV vaccines licensed and distributed in the country. This context will help readers interpret the observed low adult vaccination rates and compare them against the vaccine introduction timeline and the age range of participants.

**Our response:** Thanks for this suggestion. In the revised paper (methods) we added more contextual information including that vaccination for hepatitis B was introduced into the national vaccination programme in 2005 with 3 doses being provided as part of a pentavalent vaccine (DPTHIbHepB) administered at 6, 10 and 14 weeks of age. This is provided free of charge and financed by the Ministry of Health in collaboration with its cooperative partners. However, universal or targeted birth-dose vaccinations are not currently provided to reduce the risk of mother to child transmission but are being planned for future rollout as part of the HBV elimination 2030. The birth dose in addition to HBV immunoglobulins are procured at a cost that is usually prohibitive by parents identified by clinicians as at risk for transmission, further hampering elimination of mother to child transmission efforts. Adult vaccination at no cost is not currently available although the current national health strategy advises that all at risk individuals such as health workers should be vaccinated. Vaccine is available in the private sector but reaches few adults.

2 - In the second paragraph, the authors refer to a "third-generation" HBV vaccine. For clarity, especially for readers less familiar with HBV vaccine technology, I recommend adding a brief explanation of how third-generation vaccines differ from earlier formulations.

**Our response:** The following statement has been added to paragraph two:

The third generation of hepatitis B vaccines are recombinant vaccines that contain additional surface antigens (preS1, pre-S2 and S), compared to earlier generations that only contained S antigens which can provide a more robust immune response even from non-responders.

3 - Currently, the Introduction lacks details about the demographic and risk-group profile of participants. Since the Results section reveals that the cohort consisted mostly of young women (median age 32) from three specific high-risk groups (HCWs, PLWH, and contacts), I suggest adding a short paragraph in the Introduction summarizing the target population and the rationale for focusing on these groups.

**Our response:** Thanks for allowing us to clarify. In paragraph 7 of the introduction, we altered the statement to clearly indicate our rationale for selecting the groups. The selected groups would benefit from providing the vaccination at no cost as there is currently no campaign to actively vaccinate these risk groups.

We intended to include adult populations that strongly need HBV vaccine because of increased risk (health workers, contacts) and/or increased consequences of infection (people with HIV).

Methods:

1 - In the section describing serological testing (“...blood was collected for qualitative hepatitis B core antibody (anti-HBc) testing and qualitative and quantitative hepatitis B surface antibody (anti-HBs) testing.”), please specify the exact commercial kits used for each assay, including manufacturer name, country, assay type (e.g., ELISA, chemiluminescence), and detection thresholds. This level of detail is important for reproducibility and cross-study comparability.

**Our response:** This has been added to the methods section.

Results:

1 - Including participants with prior HBV exposure (resolved infection or isolated anti-HBc) in the vaccine response analysis may bias immunogenicity interpretation due to pre-existing immune memory. The authors should present stratified results for HBV-naïve individuals to allow an unbiased assessment of primary vaccine response. A dedicated figure or table focusing on this subgroup would enhance data clarity.

**Our response:** Our objective in the project was not to establish vaccine efficacy, but rather to understand the pool of adults who need HBV vaccination in our setting. When a non-marginal population with isolated core-Ab was observed, we decided to explore the issue of anamnestic responses to help the hospital understand the best strategy to vaccinate at-risk populations (i.e., with or without serological antibody screening first). HBV-naïve individuals were not tested for immune response; therefore, we unfortunately cannot provide the table requested by the reviewer.

Discussion:

1 - The absence of serological monitoring after the second and third vaccine doses represents a limitation, as acknowledged by the authors. Without post-series serology, it is not possible to determine whether initial non-responders developed protective anti-HBs titers after completing the full vaccination schedule. I recommend that the authors more prominently highlight this limitation and discuss its implications for interpreting the vaccine efficacy data within the studied population.

**Our response:** We agree and have more strongly explained that our exploration of non-response was exploratory, and more data are needed. The limitations of the study are included in the last 2 paragraphs of the manuscript and an additional statement to cautiously interpret the limited series will be added especially in lieu of cccDNA and its possible contribution immunogenicity to complement reviewer one’s comment.


Overall, this manuscript addresses a critically under-researched topic in HBV control efforts in Africa. The suggested clarifications and stratifications would further strengthen the paper and its contribution to the field.

Reviewer #3: General Comments and Commendation

I commend the authors for presenting original field data on HBV sero-status, vaccination uptake, and response among high-risk adult populations in Lusaka, Zambia. This is an important and timely contribution to the literature on hepatitis B prevention and control in sub-Saharan Africa. The manuscript is generally well-written, with well-articulated study objectives, design, and findings.

Major Review Comments for Improvement

1. Title and Abstract
The title is appropriate, and the abstract is comprehensive. However, I would suggest the following for an improved version of the abstract.

a. Review this sentence for clarity: “Low vaccine completion, despite vaccine access and transportation support, was striking, and underscores the need for integrated behavioral science approaches...”

**Our response:** Comma after striking will be removed for clarity: “*Low vaccine completion, despite vaccine access and transportation support, was striking and underscores the need for integrated behavioral science approaches...”*

b. The interquartile range (IQR) for median age is not specified (IQR (x, y)). Update with actual values for “x” and “y” to ensure transparency and reproducibility.

**Our response:** This was a typographical error as and will be corrected. It is found in the results. IQR will be indicated- IQR, 26.7-43.8.

c. There is no mention of sampling method (e.g., convenience, purposive) or timeframe of recruitment. I recommend you briefly mention the recruitment method and study duration.

**Our response:** The sampling method was convenience sampling and recruitment dates were from 3^rd^ October 2022 to 8th December 2023 which is indicated in the methods section.

d. Add one comparative statement to contextualize the findings from the results.

**Our response:** In the revised manuscript discussion section, we cite another report from Zambia, where 62.1% of health workers were anti-HBc positive. In that study, only 50.4% of eligible participants (241/478) completed the 3-dose vaccine series.

e. Change the sub-heading, “Discussion” to “Conclusion”

**Our response:** In the manuscript we have used Discussion to align with PLOS One guideline, but in the abstract we use Conclusion~~.~~

2. Introduction
The introduction provides a strong foundation for this article, but I suggest you explicitly state the study objectives or significance of the study in the final paragraph of the introduction.

**Our response:** These shall be added at end of the last paragraph of the introduction.

3. Methods

This section has a clear description of participant groups and serological testing strategy. However, it will benefit from addressing the following observed limitations:
a. The sampling technique is not abundantly clear whether it was convenience sampling or not? If it is ‘convenience sampling,’ then I suggest you address the implications for generalizability. You may also include sample size justification.
b. Provide more details about the statistical methods used. Specify the tests used for comparisons and state if there were any adjustments for confounders made for age, and sex, beyond what is currently written in this article?

**Our response:** Convenience sampling method was used. No adjustment of confounders was made. We have edited the methods to provide more details on the study design and statistical methods used. In the discussion we listed our sampling approach as a limitation to generalizability.

4. Results

The results section provides important insights into HBV immunity gaps and low vaccine series completion in adults. The report on anamnestic response to first vaccine dose is a novel and valuable finding. However, I strongly recommend the following adjustments:
a. The use of terms such as “resolved infection,” “isolated core antibody,” and “anamnestic response” should be consistently and clearly defined earlier in the methods/results.

**Our response:** We understand this comment and it is clear that one of the challenges in the hepatitis B field is the shear amount of terminology. To provide clarity, we defined isolated core antibody in paragraph 5 and in the methods section. In the revised paper, resolved infection is clearly defined in the methods/results. Anamnestic response was defined in the abstract, the methods and results as well as in the table 3.

b. The proportion who showed anamnestic response should be accompanied by denominators and confidence intervals, where possible.

**Our response:** This was an exploratory aspect with only 20 participants included. However, confidence intervals have been included in the new table.

c. Summarily, it would be nice to include stratified tables for (a) immunity status by subgroup, (b) vaccine uptake by subgroup, and (c) anamnestic response among isolated core antibody group. Figure 1 already addressed the need for visual aids or bar graphs for completion rates.
**Our response:** The revised table 1 displays immunity and vaccine uptake by subgroup. For anamnestic responses, we only evaluated it in 18 individuals. Their details are shown in Table 3.
5. Discussion

The discussion section impressively contextualizes the findings well. It also draws comparisons with literature from Africa and Asia, while making a strong case for policy reform and programmatic response.

However, I recommend the following areas for Improvement:
a. Even when clear sub-headings are not applied, the discussion could benefit from tighter organization addressing the following key area: Immunity Gaps, Vaccine Uptake, Anamnestic Response, and Policy Implications. Give it a thought.

**Our response:** We have opted not to give subheadings to the discussion because that is not customary for PLOS One articles in our experience.

b. Although the statement, “We believe these results support ‘blind HBV vaccination’ in Africa” is significant, it should be more rigorously supported with caveats. For example, cost-effectiveness and risk of unnecessary vaccination should be briefly mentioned.

**Our response:** A caveat will be added in the discussion related to increased cost especially where several health priorities are competing for limited funding in low resource settings but also to weigh in on the cohort effect with an obviously diminishing rate of HBV infection due to the incorporation of it in the under 5 vaccine schedule indicating a finite adult population driving the disease that could be targeted for mass blind vaccinations-where the population is skewed to young people who may already be protected.

c. The rationale for comparing with studies from Asia is understandable, but more attention should be given to regional differences in HBV epidemiology and health system context.

**Our response:** Thanks. We have worked to increase attention to HBV in African context in the revised paper.

d. Summarily, I recommend expanding on implementation science considerations (e.g., what behavior change strategies might improve vaccine completion? How could digital tools or community health workers help?). Discuss how the study informs WHO guidance or Zambia’s national HBV policy.

**Our response:** In the revised manuscript discussion section, we included priorities for the Ministry of Health of Zambia in elimination efforts and also tied this to concerns relating to cost.
6. Conclusion

The conclusion is sound, but it should be more specific about the next steps for research and implementation. For instance, consider recommending (1) integration of adult HBV vaccination into existing ART or ANC services, (2) operational research into vaccine adherence strategies, and (3) expanded national serosurveys.

**Our response:** As suggested, in the discussion section, we have added more on what is next in terms of research and implementation.

7. References

The references are current and appropriate. It also showed a strong representation of WHO guidance and peer-reviewed African studies. However, kindly review and address the inconsistent or problematic references. For example:

a. Reference numbers 2, 4, 5, 7, 8, 9, 10, 14,15, 16, 17, 18, 19, 24 (check the authors initials), 27 (incomplete author list, crosscheck the source to be sure), and 30, either lack full citation details, have capitalization concerns, missing volume issue or pages, or has concerns with authors’ initials.

**Our response:** Corrections have been made to references where appropriate. Thank you for pointing this out.

b. Crosscheck, it looks like reference number 10 is repetitive or similar to reference number 1.

**Our response.** We have checked as suggested. Reference 1 is a WHO reference detailing global HBV prevalence, reference 10 is related to African prevalence from a systematic review.

c. Crosscheck, it looks like reference number 21 is repetitive or similar to reference number 13.

**Our response.** We have checked as suggested. They are also different.

8. General Minor Comments

a. For line-editing to improve grammar and flow, consider changing “lower rates of infection HBV among HCWs” to “lower HBV infection rates among HCWs”.
b. Consider replacing non-academic phrases like “strikingly few” with more measured terms like “a low proportion.”
c. I suggest that you clarify in the discussion whether “blind vaccination” would apply only to high-risk adults or to general adult populations.
d. Review and ensure that acronyms are consistently defined at first use (e.g., PLWH, HCW, anti-HBc, HBsAg).
e. I suggest you specify if this was a cross-sectional or longitudinal study, especially as it seems like the language is occasionally inconsistent.
e. Take some time to read through the entire work again and carry out some editing and rephrasing, to enhance clarity and flow of the article.

**Our response:** We have made amendments where appropriate.
