# Supplementary material for: Hepatitis B immunity and vaccine completion among adults at increased risk for hepatitis B infection in Zambia
Source: PLoS One. 2026 Feb 26;21(2):e0339690. doi: 10.1371/journal.pone.0339690 (PMC12944761; doi:10.1371/journal.pone.0339690)
Supplement: Attachment — Submitted filename: Responses to reviewers.docx [file pone.0339690.s004.docx]

**Responses to reviewers**

1. If the authors have adequately addressed your comments raised in a previous round of review and you feel that this manuscript is now acceptable for publication, you may indicate that here to bypass the “Comments to the Author” section, enter your conflict of interest statement in the “Confidential to Editor” section, and submit your "Accept" recommendation.

Reviewer #1: (No Response)

Reviewer #2: All comments have been addressed

Reviewer #3: All comments have been addressed

2. Is the manuscript technically sound, and do the data support the conclusions?

The manuscript must describe a technically sound piece of scientific research with data that supports the conclusions. Experiments must have been conducted rigorously, with appropriate controls, replication, and sample sizes. The conclusions must be drawn appropriately based on the data presented.

Reviewer #1: Yes

Reviewer #2: Yes

Reviewer #3: Yes

3. Has the statistical analysis been performed appropriately and rigorously?

Reviewer #1: I Don't Know

Reviewer #2: Yes

Reviewer #3: Yes

4. Have the authors made all data underlying the findings in their manuscript fully available?

The PLOS Data policy requires authors to make all data underlying the findings described in their manuscript fully available without restriction, with rare exception (please refer to the Data Availability Statement in the manuscript PDF file). The data should be provided as part of the manuscript or its supporting information, or deposited to a public repository. For example, in addition to summary statistics, the data points behind means, medians and variance measures should be available. If there are restrictions on publicly sharing data—e.g. participant privacy or use of data from a third party—those must be specified.

Reviewer #1: Yes

Reviewer #2: Yes

Reviewer #3: Yes

5. Is the manuscript presented in an intelligible fashion and written in standard English?

PLOS ONE does not copyedit accepted manuscripts, so the language in submitted articles must be clear, correct, and unambiguous. Any typographical or grammatical errors should be corrected at revision, so please note any specific errors here.

Reviewer #1: Yes

Reviewer #2: Yes

Reviewer #3: Yes

6. Review Comments to the Author

Please use the space provided to explain your answers to the questions above. You may also include additional comments for the author, including concerns about dual publication, research ethics, or publication ethics. (Please upload your review as an attachment if it exceeds 20,000 characters)

Reviewer #1: The authors have partially addressed some of my specific questions and provided additional information. However, there remains a lack of detail and clarity in the abstract and throughout the manuscript.

Since this is a paper about HBV vaccination, the authors should at least include information about the composition of the HBV vaccine used. The issue is that in their new introduction, the authors wrote that “Since the first hepatitis vaccine in

1981[5], several iterations have been developed to optimise immunogenicity and

safety. The third (current) generation of hepatitis B vaccines are recombinant,

containing multiple additional surface antigens (preS1, pre-S2 and S), whereas earlier

vaccines had only contained S antigens, which can provide a more robust immune

response even from non-responders [6].”

The authors seem to suggest that their vaccine now contains the Large envelope protein (PreS1, PreS2 and S), something that I sincerely doubt. Currently, the most widely used vaccine worldwide contains only S, not PreS1 and PreS2.

PreS1/PreS2/S vaccines have been developed; they are highly immunogenic and do not require the full three-dose course, but they are also more expensive (this vaccine needs to be produced in mammalian cells). The authors used the BEVAC rDNA Hepatitis B vaccine (Biological E Limited, India), but I am unable to determine whether this vaccine contains only S or also PreS1/PreS2/S. While this information might not be essential for writing a report about “vaccine acceptance in the population,” since the authors claim that “full course of HBV vaccination should be implemented in individuals with positivity of anti-HBc, " it is important to understand exactly which vaccine was used and which adjuvant it contained.

Additionally, because the analysis of the vaccine's immunogenicity in anti-Hbc+ individuals was conducted in only 18 patients, the data are at best preliminary and should not be presented as a major result of this report.

The vaccine used in the study was indeed a 2^nd^ generation hepatitis b vaccine and as such contained the S antigen as it is the most widespread. This has been added to the introduction as well as the methods section.

A caveat has been made in the discussion section to further highlight the preliminary nature of the results shown from the anti HBc+ individuals.

AN addition

Reviewer #2: The authors have satisfactorily addressed the revisions requested during my first review and have provided clear clarifications to the points previously raised. I now consider the manuscript appropriate for publication.

Reviewer #3: (No Response)

7. PLOS authors have the option to publish the peer review history of their article (what does this mean?). If published, this will include your full peer review and any attached files.

If you choose “no”, your identity will remain anonymous but your review may still be made public.

Do you want your identity to be public for this peer review? For information about this choice, including consent withdrawal, please see our Privacy Policy.

Reviewer #1: No

Reviewer #2: No

Reviewer #3: Yes: Ojore Godday Aghedo
